# Impact of CAD-deficiency in flax on biogas production

**DOI:** 10.1007/s11248-015-9894-4

**Published:** 2015-07-16

**Authors:** Magdalena Wróbel-Kwiatkowska, Sławomir Jabłoński, Jakub Szperlik, Lucyna Dymińska, Marcin Łukaszewicz, Waldemar Rymowicz, Jerzy Hanuza, Jan Szopa

**Affiliations:** Department of Biotechnology and Food Microbiology, Wrocław University of Environmental and Life Sciences, Chełmońskiego Str. 37/41, 51-630 Wrocław, Poland; Department of Biotransformation, Faculty of Biotechnology, University of Wrocław, F. Joliot-Curie 14a, 50-383 Wrocław, Poland; Faculty of Biotechnology, University of Wrocław, Przybyszewskiego 63, 51-147 Wrocław, Poland; Department of Bioorganic Chemistry, Faculty of Engineering and Economics, Institute of Chemistry and Food Technology, Wrocław University of Economics, Komandorska 118/120, 50-345 Wrocław, Poland; Department of Genetics, Plant Breeding and Seed Production, Wrocław University of Environmental and Life Sciences, pl. Grunwaldzki 24A, 53-363 Wrocław, Poland

**Keywords:** *CAD* gene, Transgenic flax, Biogas production, FTIR (Fourier transform infrared spectroscopy), Shives

## Abstract

Global warming and the reduction in our fossil fuel reservoir have forced humanity to look for new means of energy production. Agricultural waste remains a large source for biofuel and bioenergy production. Flax shives are a waste product obtained during the processing of flax fibers. We investigated the possibility of using low-lignin flax shives for biogas production, specifically by assessing the impact of CAD deficiency on the biochemical and structural properties of shives. The study used genetically modified flax plants with a silenced *CAD* gene, which encodes the key enzyme for lignin synthesis. Reducing the lignin content modified cellulose crystallinity, improved flax shive fermentation and optimized biogas production. Chemical pretreatment of the shive biomass further increased biogas production efficiency.

## Introduction

Flax (*Linum usitatissimum L.*) is an annual plant with a very long history of cultivation worldwide. There are two products: fibers, which are used in textiles and composites (Gredes et al. 2012; Wróbel-Kwiatkowska et al. 2012) and oil, which is rich in unsaturated fatty acids and has benefits for human health. Biotechnological methods have been used to generate novel flax plants that are more resistant to pathogens (Wróbel-Kwiatkowska et al. 2004), are enriched with flavonoids and phenolic acids (Lorenc-Kukuła et al. 2007) and have improved fiber quality.

Fiber quality improvements have been accomplished using two different strategies: the synthesis of exogenous thermoplastic polymer (PHB) in the flax fibers (Wróbel-Kwiatkowska et al. 2007a); and the reduction of the endogenous polymer (lignin) content in the flax fibers (Preisner et al. 2014; Wróbel-Kwiatkowska et al. 2007b). Lignin consists of compounds from phenylpropanoid pathway (coniferyl-, sinapyl- and p-coumaryl-alcohols). It is not a desirable constituent of fibers as it is responsible for mechanical resistance and hardness. The absence of lignin in cotton fibers is the reason for the textile industry’s greater reliance on cotton.

Using a previously described method, we generated flax plants with constitutive repression of the *CAD* gene, which codes for the key enzyme for lignin synthesis, cinnamyl alcohol dehydrogenase (Wróbel-Kwiatkowska et al. 2007b). Our aim was to use flax shives derived from these plants as the substrate for biogas production.

Flax shives are a waste product of the fiber extraction process. For each ton of flax fiber, 2.5 tons of shives are produced (Ross and Mazza 2010). They are mainly used as a component of packaging materials, but their use for energy production and as fuel was recently proposed (Kymalainen et al. 2004).

Two factors negatively affect the efficiency of agricultural waste usage for biogas production: the presence of lignin, which exhibits high resistance to both chemical and enzymatic degradation; and cellulose crystallinity (Taherzadeh and Karimi 2008). Many protocols for lignocellulose pretreatment were developed to improve plant biomass as a substrate for biofuel and biogas production (Hendriks and Zeeman 2009), including mechanical (milling), physical (steam explosion, radiation), chemical (acids, bases or solvents) and biological methods (enzymes or fungi; Teghammar et al. 2014).

For our study on biogas production, as well as genetic modification, we used a chemical method: pretreatment with an aqueous solution of sulfuric acid or sodium hydroxide or with water. The biochemical composition of the genetically modified flax shives was assessed and a structural analysis of the tested shives was performed to determine the effects of CAD deficiency on their properties and potential applications.

## Materials and methods

### Raw material

Flax shives were obtained from CAD27 plants and unmodified flax plants of the cultivar Nike, which acted as the control (Wróbel-Kwiatkowska et al. 2007b). The plants had been cultivated in the field for 4 months and then retted using the dew method (Wróbel-Kwiatkowska et al. 2007a). Flax straw was processed into fibers and shives in mechanical decortications. The separated shives were used for this study. For all the experiments, the shives were pretreated with 2 M NaOH, 1 M H_2_SO_4_ or water for 72 h at 37 °C. Then the samples were centrifuged, washed with distilled water and used for anaerobic biodegradation tests.

### Anaerobic biodegradation tests

The anaerobic biodegradation tests were prepared in 120 ml serum bottles. Fifty ml of the inoculum and 0.5 g of the shive samples were placed in each bottle. The substrate was omitted from the control bottles. The inoculum material was obtained from a laboratory anaerobic reactor fed with cow manure and had the following parameters: pH 7.04, 400 mg/l [NH_4_^+^], 5.14 % DW and 3.56 % volatile solids.

In the next step, the air was removed from the bottles by flushing them with nitrogen gas. The digestion test took place at 37 °C. Once every 24 h for a 21-day period, the samples were stirred and then gas measurements were taken via water displacement (Kida et al. 2001). All of the samples were prepared in triplicate.

The amount of biogas produced from biomass was calculated as the difference between the production in the sample bottles and the production in the control bottles. Biogas volumes were calculated for the standard state (10^5^ Pa, 298.15 K), pH was measured with a CyberScan pH5500 pH/Ion Meter (Eutech Instruments), and the ammonium ion concentration was measured with an ion-selective electrode (Eutech Instruments) according to the manufacturers’ instructions. The dry weight and volatile solids were determined as described previously (Clesceri et al. 1998).

### Determination of the lignin content in the flax shives

For lignin analysis, the acetyl bromide method was used as described previously (Iiyama and Wallis 1990). Using a previously described procedure, lignin was isolated from two types of flax shives, derived from the CAD27 flax plants and the unmodified, wild-type plants (Wróbel-Kwiatkowska et al. 2009).

### Determination of cellulose content in flax shives

The cellulose content was measured using the colorimetric method with anthrone reagent (Updegraff 1969). Shive samples were prepared and cellulose measured as described previously (Wróbel-Kwiatkowska et al. 2009).

### Analysis of pectin content in flax shives

For the pectin analysis, the modified method of Melton and Smith was used (Melton and Smith 2001). The shive samples were washed with 96 % ethanol (100 °C), and after centrifugation (5000*g*, 5 min) the pellet was washed with 80 % ethanol (80 °C) and treated with mixture of chloroform and methanol (1:1, v/v). Then the samples were centrifuged again (5000*g*, 5 min) and the remaining pellet was washed with acetone and centrifuged as before. The dried pellet (at 37 °C) was frozen and weighed. Acidic hydrolysis was performed with concentrated sulfuric acid, and then the samples were stirred for 5 min on ice. Next, the samples were diluted with water and centrifuged (2000*g*, 10 min). Supernatants were taken to measure pectin using a modified version of the biphenyl method described in Blumenkrantz and Asboe-Hansen (1973). A 4 M sulfamic acid/potassium sulfamate solution was added to each sample and mixed. Then 75 mM sodium tetraborate in sulfuric acid was added and again mixed. The samples were incubated at 100 °C for 20 min and cooled on ice for 10 min. Then 0.15 % m-hydroxy-biphenyl in 0.5 % NaOH was added and mixed. In samples that were incubated for 10 min at room temperature, the pectin content was measured at 525 nm and galacturonic acid was used to prepare a calibration curve.

### IR studies

The IR spectra of the shive samples were measured in the spectral range 350–4000 cm^−1^ using a FT-IR NICOLET 6700 spectrometer as described earlier (Dymińska et al. 2012). The mathematical processing of the measured spectra was performed using the computer program ORIGIN 7.5. Lorentzian distribution function was used for data fitting and the fitting parameter χ^2^ was of the order 10^−6^. The crystallinity index was estimated as the ratio of bands at 2900 and 1370 cm^−1^ (Langan et al. 1999; Nishiyama et al. 2003).

## Results

### Biogas production

**Fig. 1:**
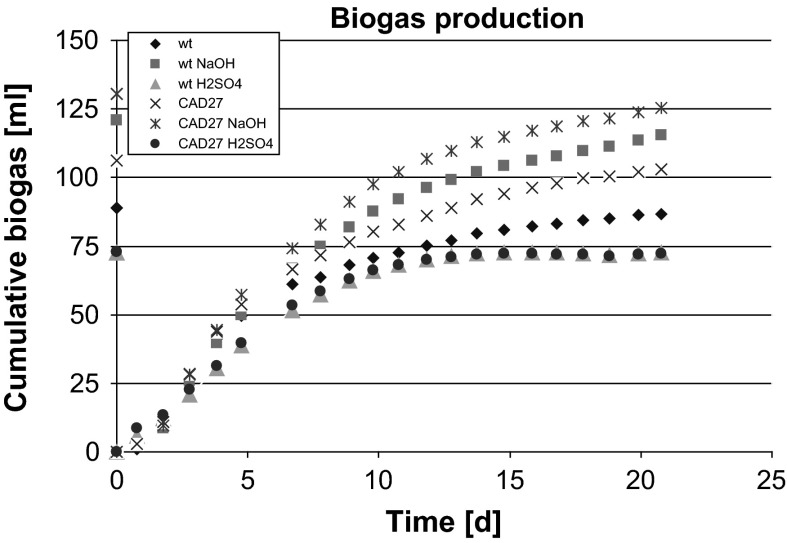
Biogas production. The anaerobic biodegradation tests were prepared as it was specified in the “Materials and methods” section. The inoculum material is characterized in Table 1. All of the samples were prepared in triplicate

Flax shives were used as the substrates for microbial fermentation and biogas production. The shives from the unmodified control plants and genetically modified flax plants were pretreated with acid, alkali or water and supplemented with inoculum material obtained from a laboratory anaerobic reactor fed with cow manure. The biogas production was measured as described in the “Materials and methods” section. The data are presented in Fig. 1.

The highest level of biogas was noticed for shives derived from CAD27 plants pretreated with sodium hydroxide. It was about 7 % higher than for unmodified shives after alkalization. A lower level of cumulative biogas was produced when shives from CAD27 plants were pretreated with water and then added into the inoculum, but the amount of biogas was still higher than for shives from control plants pretreated with water. The lowest amount of biogas was produced when shives pretreated with sulfuric acid were used. It was observed for shives from both control and transgenic plants.

### Biochemical analysis of chemically treated shives

**Fig. 2:**
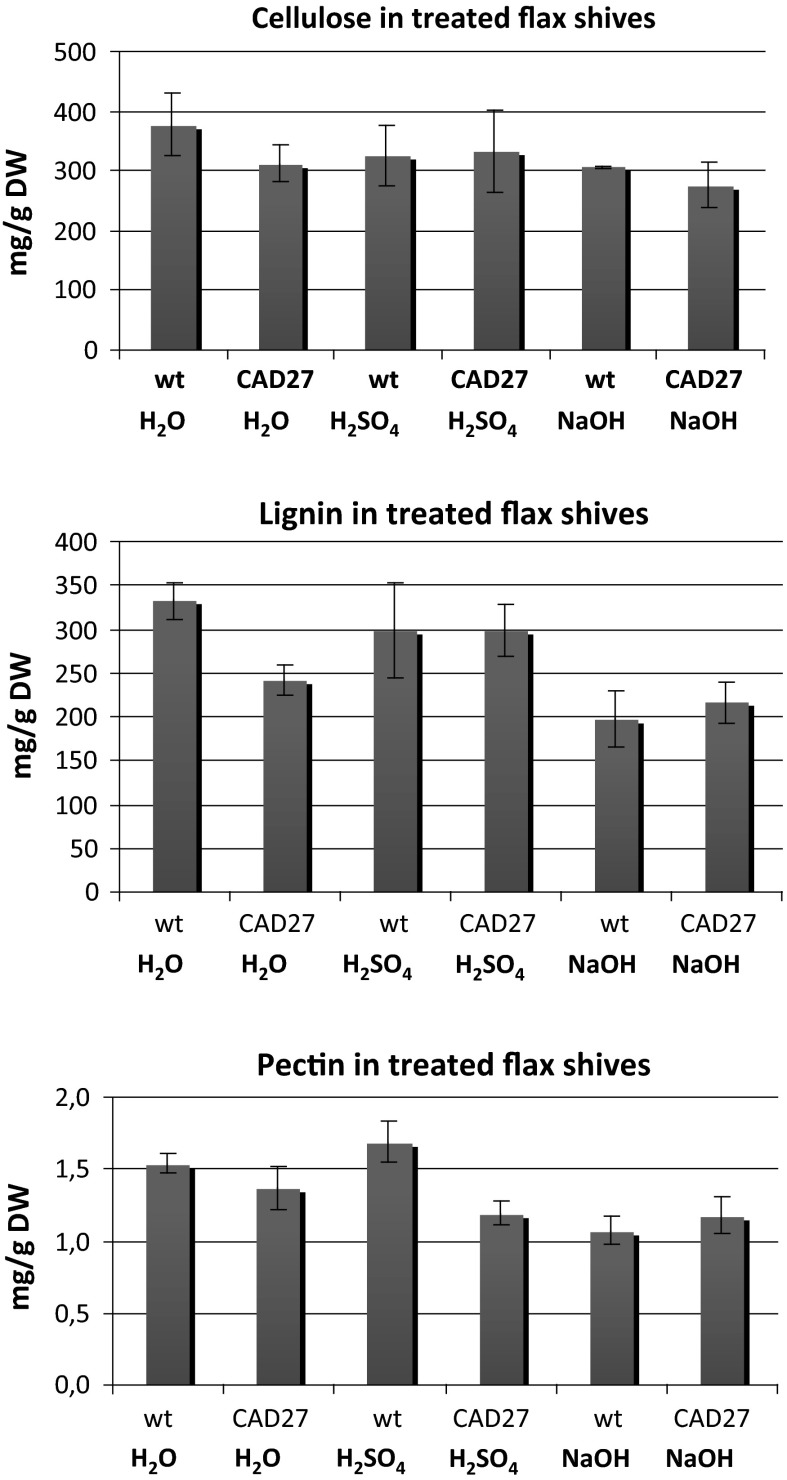
The lignin, cellulose and pectin contents in shives from transgenic flax (CAD27) and control, unmodified shives (wild-type; wt) treated with water, H_2_SO_4_ or NaOH. The measurements were done as it was described in the “Materials and methods” section. The mean value ± SD is presented (n = 3–6)

To determine the reason for the higher level of biogas produced when transgenic shives were used, we analyzed the chemical composition of the shives (Fig. 2). The levels of cellulose, lignin and pectin were lower in shives from the transgenic plants than from the control plants. Thus, the polymer profile of the shives is different than that of the fibers, in which CAD-deficiency caused an accumulation of cellulose and pectin (Preisner et al. 2014).

The degree of lignification is a parameter that determines the usage of plant biomass for energy production. Reduction of lignin results in easier access to cellulose and improves its degradability. Lignin content in the shives from CAD27 plants pretreated with water was about 27 % lower than for shives from the control plants pretreated in the same way (Fig. 2). The amount of lignin in shives from both plant types pretreated with sulfuric acid was quite similar.

The highest reduction in lignin level was noticed for shives from both control and transgenic plants treated with sodium hydroxide. The highest levels of accumulated biogas were also measured for these shives. This confirmed that the lignin content negatively correlates with biogas production. It is interesting that the lignin levels of the two types are essentially the same, but the biogas production level is higher when shives from transgenic plants were used. This suggests that another parameter strongly affects biogas production.

We performed biochemical analyses of other compounds in the shives. Cellulose constitutes about 50 % of their biochemical composition (Ross and Mazza 2010). The level of cellulose was reduced in shives from transgenic flax pretreated with water in comparison to pretreated control (Fig. 2). Pretreatment with sulfuric acid did not lead to a difference in the cellulose levels between the shives from transgenic and control plants. However, pretreatment with alkali reduced the cellulose level in the shives from both plant types and in the transgenic plants, the reduction was higher than for the control. It should be noted that the changes in cellulose content upon shive pretreatment did not reflect the change in biogas production.

Pectin is another component of shives. It inhibits the access of the enzymes to cellulose (Xiao and Anderson 2013; Park et al. 2010). On the other hand pectin may confer hydrophilic character and improve the availability of cellulose from the shives to the inoculum. The level of pectin was reduced in shives from CAD27 plants compared to the control (Fig. 2). The lowest pectin level was observed when alkali treatment was applied. This lower pectin level might be the reason for the easier access of enzymes from the inoculum to the cellulose.

### Spectral analysis of the shives

**Fig. 3:**
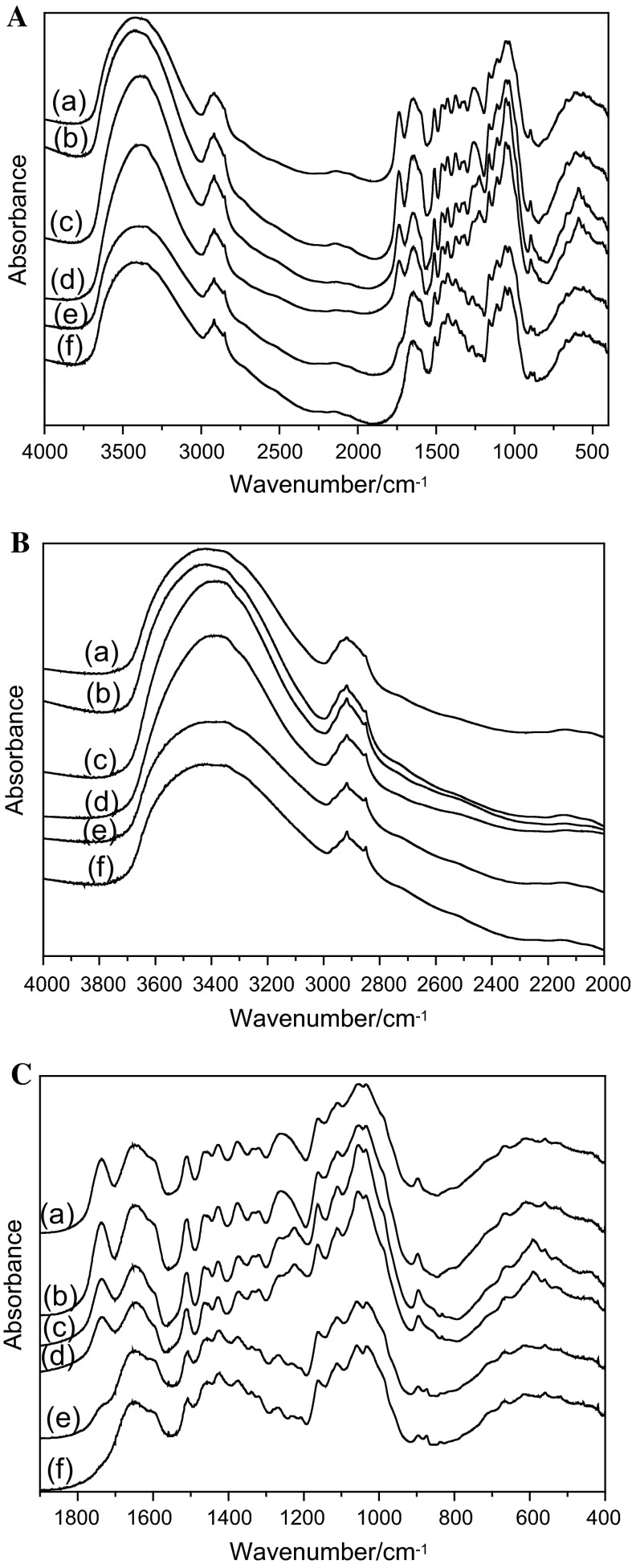
**A** IR spectra of WT+H_2_O (*a*), CAD27+H_2_O (*b*), WT+H_2_SO_4_ (*c*), CAD27+H_2_SO_4_ (*d*), WT+NaOH (*e*) and CAD27+NaOH (*f*) shives. **B** IR spectra in the region 4000–2000 cm^−1^ of WT+H_2_O (*a*), CAD27+H_2_O (*b*), WT+H_2_SO_4_ (*c*), CAD27+H_2_SO_4_ (*d*), WT+NaOH (*e*) and CAD27+NaOH (*f*) shives. **C** IR spectra in the region 1850–400 cm^−1^ of WT+H_2_O (*a*), CAD27+H_2_O (*b*), WT+H_2_SO_4_ (*c*), CAD27+H_2_SO_4_ (*d*), WT+NaOH (*e*) and CAD27+NaOH (*f*) shives

Spectroscopic analyses of the shives and the arrangement of the basic constituents of the shives were performed using the FTIR method. The IR spectra of shives from the control and transgenic plants pretreated with water, acid and alkali are presented in Fig. 3. Four characteristic ranges were detected: 2000–4000, 1200–1800, 900–1200 and 400–900 cm^−1^. The main contours are similar to those reported earlier for flax (Blackwell et al. 1970; Jähn et al. 2002; Schwanninger et al. 2004). Absorption intensity at 3400 cm^−1^, which has previously been described as characteristic for free hydroxyl groups (Wróbel-Kwiatkowska et al. 2009), differed in the analyzed shives: the IR bands in the OH stretching range could be deconvoluted into five Lorentzian components (Table 1).

**Table 1:** Wavenumbers (ν) of the Lorentzian components derived for the 3000–3700 cm^−1^ range for the control (WT) and transgenic (CAD27) flax shives

WT+H_2_O	WT+H_2_SO_4_	WT+NaOH	CAD27+H_2_O	CAD27+H_2_SO_4_	CAD27+NaOH
ν	ν	ν	ν	ν	ν
3614	3591	3608	3616	3598	3606
3551	3507	3529	3559	3525	3521
3467	3396	3416	3461	3418	3404
3364			3330		
3242	3269	3281	3206	3280	3279
	3006	3120		3002	3125

Intramolecular 2-OH···O-6 hydrogen bonds (band at 3470–3440 cm^−1^) in control and transgenic plant shives pretreated with H_2_SO_4_ and NaOH are longer and weaker (appear at shorter wavenumbers) than those in shives treated with H_2_O (Table 1). Intermolecular 6-OH···O-3′ hydrogen bonds in cellulose (band at 3280–3200 cm^−1^) are stronger and shorter in shives treated with H_2_SO_4_ and NaOH. It results from shifts of bands positions toward longer wavenumbers for these samples in comparison to shives treated with water (Carrillo and Colom 2004; Schwanninger et al. 2004; Dai and Fan 2011). A new band (at 3130–3000 cm^−1^) is formed in the mentioned shives. It is typical for an intermolecular 2-OH···O-2′ hydrogen bond (Oh et al. 2005), while the band for an intramolecular 3-OH···O-5 (at 3380–3330 cm^−1^) hydrogen bond is absent in mentioned shives. The obtained data might suggest changes in cellulose arrangement in shives from the transgenic plants and a higher tendency for the pyranoid rings to rotate after acid and alkali pretreatment.

The crystallinity index (I_cr_) of cellulose is an important factor that characterizes the degree of crystallinity of cellulose. It expresses the ratio between crystalline structures and amorphous structures in cellulose. I_cr_ negatively correlates with both access to cellulose for degrading enzymes and the hydrophilic properties of cellulose. The hydrophilic properties of cellulose are responsible for the degree of mixing with other materials (Kirk et al. 1968), including the inoculum used for biogas production. The crystallinity index was measured in the shives (Table 2) and found to have higher values for the untreated and treated control shives than for the respective shives from the transgenic plants.

**Table 2:** The crystallinity index (I_cr_) estimated for control and transgenic shives

Type of chemical modification of shives	I_CR_ for control shives	I_CR_ for transgenic shives
Treatment with H_2_O	15.0	11.78
Treatment with H_2_SO_4_	7.12	6.0
Treatment with NaOH	13.0	9.0

The parameter was calculated as the intensity ratio of the bands at 2900 and 1370 cm^−1^

Although the lowest crystallinity index value was found for the shives after acid treatment, they were not a sufficient substrate for fermentation and biogas production. The reason might be the amount of lignin, which was about 50 % higher than that in shives treated with alkali. This shows that lignin amount is the key parameter that influences the degradability of biomass.

## Discussion

The aims of this study were to estimate the effect of CAD reduction on the biochemical and structural properties of flax shives and to analyze the impact of genetic and chemical modifications on cumulative biogas production.

The main problem with using agricultural waste as bioenergy substrates is the presence of lignin, which is the most resistant compound of the plant cell wall (Walton 1994). Reducing the lignin level in plants is the purpose of many studies (Chen and Dixon 2007; Li et al. 2008, Hisano et al. 2009).

In this study, we used flax shives, which are a waste product from fiber production, as a biogas substrate. They were obtained from transgenic plants with a reduced lignin level: about 30 % lower than in the control shives. The lignin content was further decreased by chemical pretreatment with acid, alkali or water. It should be noted that the reduction in lignin amount caused by alkalization was higher for the control shives than for the transgenic shives.

The highest amount of cumulative biogas was detected when the substrate was shives from transgenic CAD27 plants pretreated with alkali, although they showed almost the same level of lignin as the control shives after alkalization. Thus, we expected that there must be different agent that affects biogas production and cellulose fermentation.

The structural characteristics of shives were assessed. We found that the crystallinity index in the control shives was higher than that for shives from CAD27 plants, even those treated with alkali or water. A high cellulose crystallinity value results in a lower level of digestibility (Jeoh et al. 2007). Thus, the observed higher cellulose crystallinity for all the control samples might be the reason for reduced cellulose digestion by microorganisms from the inoculum.

However, other factors clearly play an important role and the relationships between parameters are complex. The highest observed difference in the amount of produced biogas was between transgenic and control shives treated with water. In this case, the amount of biogas correlated with a reduced lignin content in those shives. Thus it can be suggested that lignin reduction is the main reason for higher biogas production.

Flax shives also contain pectin. Shives derived from CAD27 plants showed 20 % lower pectin content than the controls and a further reduction was observed after alkali pretreatment. Interestingly, the control shives reached the same pectin content after treatment with NaOH, so pectin did not have a direct impact on the produced cumulative biogas, which achieved a higher value when alkali-treated shives from transgenic plants were used as a substrate.

The obtained data suggest that the efficiency of biogas production from flax shives depends mainly on the lignin amount. The similar observation was described for example for poplar and *Miscanthus*, in those plants reduction in the lignin level caused increased bioethanol production (Mansfield et al. 2012; da Costa et al. 2014). It should be pointed out that the reduction in the lignin level is advantageous when biological biodegradation by microorganisms is exploited. However, for direct combustion a decrease in the lignin could be a disadvantage, because it results in a lower energy value of plant material (Allison et al. 2010; Hodgson et al. 2010).

An additional advantage seen in our study is the decreased index of cellulose crystallinity (I_cr_) in the CAD deficient plants. The highest observed difference in the I_cr_ of cellulose between control and transgenic shives was found for shives treated with alkali, which might suggest that a decreased lignin content directly influences the degree of crystallinity of cellulose.

In this paper, we try to explain the impact of CAD silencing in flax on bioenergy production. CAD deficiency has a positive influence on cumulative biogas production. A decreased lignin level is especially important for fibrous cultivars of flax, because it positively correlates with the mechanical parameters of the fibers and thus improves the quality of fibers.

CAD deficiency is overall advantageous for the quality of flax fiber and favorable for the utilization of shives, a waste product derived from fiber processing. The lignin reduction obtained through genetic manipulation might provide a means for using the whole flax plant meaning less wasteful agricultural practices and renewable bioenergy strategies.
